# Mapping Sleeping Bees within Their Nest: Spatial and Temporal Analysis of Worker Honey Bee Sleep

**DOI:** 10.1371/journal.pone.0102316

**Published:** 2014-07-16

**Authors:** Barrett Anthony Klein, Martin Stiegler, Arno Klein, Jürgen Tautz

**Affiliations:** 1 The University of Wisconsin – La Crosse, Department of Biology, La Crosse, Wisconsin, United States of America; 2 University of Würzburg, BEEgroup, Biozentrum, Würzburg, Germany; 3 Sage Bionetworks, Seattle, Washington, United States of America; University of Arizona, United States of America

## Abstract

Patterns of behavior within societies have long been visualized and interpreted using maps. Mapping the occurrence of sleep across individuals within a society could offer clues as to functional aspects of sleep. In spite of this, a detailed spatial analysis of sleep has never been conducted on an invertebrate society. We introduce the concept of mapping sleep across an insect society, and provide an empirical example, mapping sleep patterns within colonies of European honey bees (*Apis mellifera* L.). Honey bees face variables such as temperature and position of resources within their colony's nest that may impact their sleep. We mapped sleep behavior and temperature of worker bees and produced maps of their nest's comb contents as the colony grew and contents changed. By following marked bees, we discovered that individuals slept in many locations, but bees of different worker castes slept in different areas of the nest relative to position of the brood and surrounding temperature. Older worker bees generally slept outside cells, closer to the perimeter of the nest, in colder regions, and away from uncapped brood. Younger worker bees generally slept inside cells and closer to the center of the nest, and spent more time asleep than awake when surrounded by uncapped brood. The average surface temperature of sleeping foragers was lower than the surface temperature of their surroundings, offering a possible indicator of sleep for this caste. We propose mechanisms that could generate caste-dependent sleep patterns and discuss functional significance of these patterns.

## Introduction

Maps help to integrate data in ways that clarify patterns or relationships in the lives of organisms. Mapping social phenomena can reveal the spread of disease [1], routes of migration [2], foraging paths [3], organization with respect to division of labor [4] or brood sorting [5], spatial segregation of individuals within a colony [6], or spatial dynamics of competing colonies [7]. Social insect colonies, and honey bee (*Apis mellifera* L.) colonies in particular, lend themselves well to mapping of behavior. Honey bee activity has been visualized outside the nest with respect to flight paths [8], [9], simulated flight paths relative to landmarks [10], and inside the nest for spatial organization of waggle dance information [11] and patterns generated by removal rates of comb contents [12]. Seeley [13] created maps depicting twelve of the most commonly performed tasks within a nest of honey bees. Conspicuously absent, however, are maps depicting where bees reside when *not* performing tasks. Sleep is a behavior that has never been mapped extensively across an invertebrate society, in spite of its potential ecological and evolutionary significance. Studies mapping sleep of invertebrates are limited to measurements of individual inactivity, usually within highly artificial settings (e.g., fruit fly stasis within test tubes, [14]). This is in contrast with the more extensive literature devoted to the study of vertebrate sleep sites, which has offered insight as to some functional implications of sleeping socially, especially with respect to vigilance and predator avoidance [15], [16], [17].

**Figure 1:**
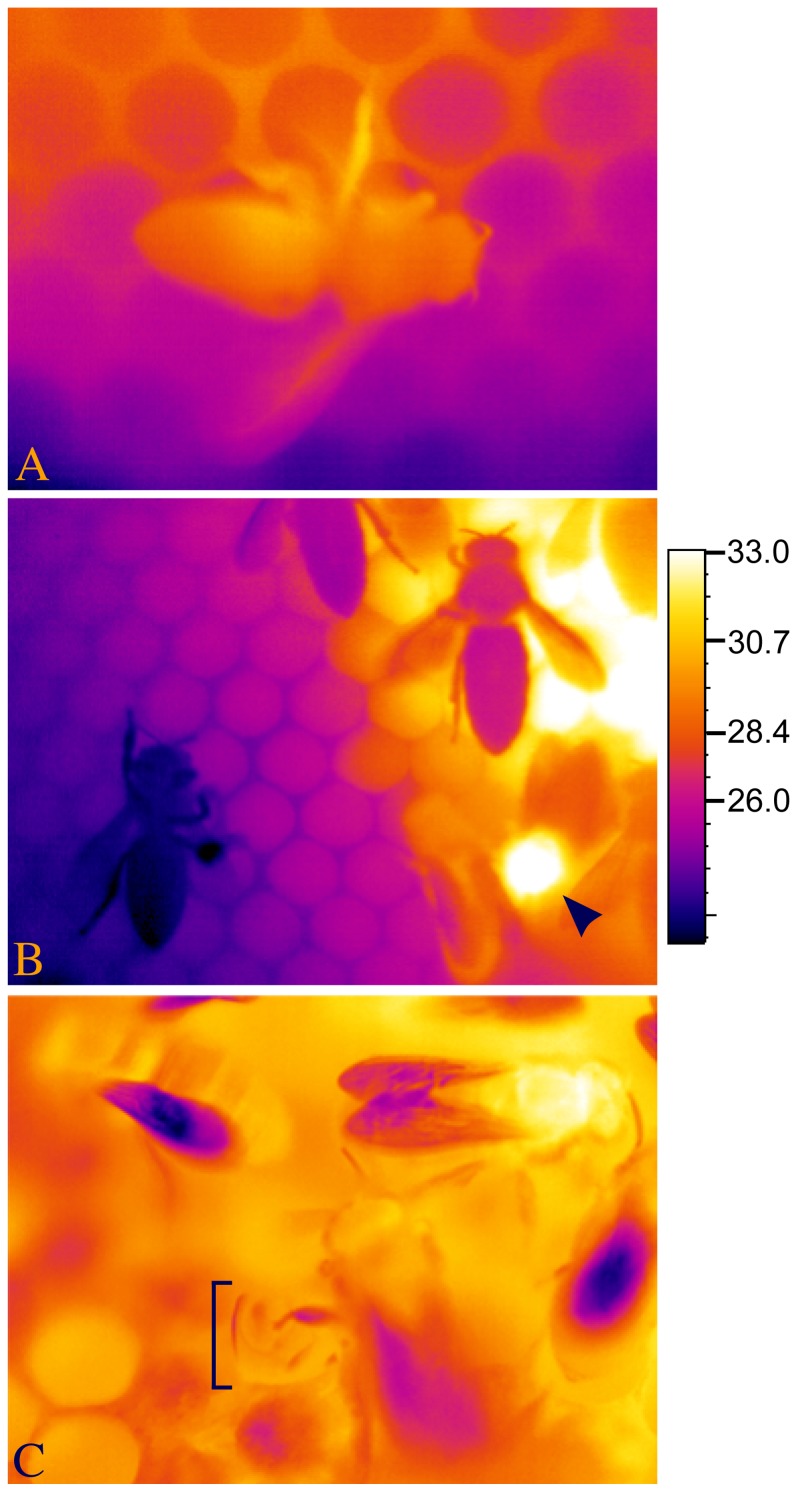
Infrared images of sleeping honey bees. (A) A sleeping honey bee outside of a cell is relatively immobile, typically hangs in the direction of gravity (in this case she hung horizontally, facing right), and discontinuously ventilates, pumping her abdomen (metasoma) in the anterior-posterior direction. This bee was exhibiting deep sleep, with extended bouts of antennal immobility. (B) Sleeping bees are affected by surrounding temperature, as with the bee on the perimeter of the hot brood comb (upper right) and the inverted bee, hanging (lower left) on the plastic window a short distance from the brood comb. For comparison, note the mobile bee at lower right with the relatively hot thorax (arrowhead). (C) Bees also slept inside cells, primarily when they were young adults. Here, framed by the bracket, is the abdomen of a sleeping bee slightly protruding from a cell (leg of neighboring bee resting on the distal tip of her abdomen), distinguished as sleeping by her discontinuous ventilatory movements. Temperatures of bees sleeping inside cells were not recorded. B.A.K. took all images with FLIR thermal cameras on non-experiment days under different ambient temperature conditions; °C temperature scale only relevant for (B), and values were adjusted for thermal camera settings (see Materials and Methods).

Sleep is defined behaviorally by a suite of characters [18], [19], [20] that have been identified in honey bees [21], [22] (Fig. 1; see Materials & Methods for operational definition). Honey bee workers typically progress through a chronological sequence of task-based castes, beginning adulthood as cell cleaners [23], [24], [25], later tending brood and queen as nurse bees, then receiving and storing nectar as food storers [26], and ultimately serving as the colony's foragers [13]. Because the different worker castes engage in tasks that have some spatial component, we hypothesized that bees belonging to different worker castes would sleep in different areas of the nest, depending in part on distance from the bustling brood comb. By sleeping while exposed to an incessantly working mass of siblings cleaning cells and tending the brood, bees could face frequent disturbance and sleep fragmentation. Following Kaiser's [21] observations of unknown caste members sleeping near the perimeter of the comb and Klein et al's [27] observations of a single forager over 24 h, we predicted that foragers sleep closer to the perimeter of the comb and away from uncapped brood cells (brood cells not yet capped by the juveniles' older sisters). In contrast, we predicted that younger castes sleep inside cells within the brood comb area.

Investigating the spatial patterns of sleep in colonies of honey bees could benefit from a visualization technique that distinguishes sleeping from non-sleeping individuals. Honey bees of unknown caste have been reported to sleep at ambient temperature at the perimeter of their nest [21] or when resting inside cells [28], and foragers have been reported to sleep within a certain range of ambient temperatures when isolated [21], [29], [30] or in unreported locations of the nest [30]. If honey bees of different worker castes sleep in different areas of the nest, is temperature, alone or in combination with nest landmarks, a good predictor for determining which honey bees are asleep? We hypothesized that ontogenetic changes and caste-dependent demands of aging honey bees would result in predictable thermal and sleep patterns across a colony's nest. We applied remote sensing thermography to map caste-dependent sleep by individually marking newly eclosed honey bees and recording their behaviors at different stages of their adult lives. Our aim was to uncover caste-dependent sleep patterns with respect to a bee's location in the nest. We identified spatial patterns among sleeping bees within their nest, and propose a possible role of thermography in further mapping sleep behavior in honey bees.

## Materials & Methods

We studied the sleep behavior, location, and surface temperatures associated with Carniolan worker honey bees (*A. mellifera carnica* Pollman, 1879) shortly after eclosion and during subsequent periods of their adult lives as the bees changed castes. We observed two colonies in separate years; differences between the studies of Colony 1 (2006) and Colony 2 (2008) are noted throughout. First, we installed a two-frame observation hive [31] in a temperature-controlled room at the bee research facility of the University of Würzburg (Würzburg, Germany, 49°46′47″N, 9°58′31″E), and allowed the hive of bees unrestricted access to the outdoors, where bees freely foraged during the day. We introduced 49 recently eclosed, individually-marked worker bees to Colony 1 on 2 June 2006 and 49 to Colony 2 on 14 August 2008. The bees had been extracted within hours of eclosing from a brood comb placed in a 35°C incubator (Colony 1), or had been extracted directly from six different outdoor hives (Colony 2). We individually marked the dorsal mesosoma (referred elsewhere as thorax), and dorsal and ventral metasoma (referred elsewhere as abdomen) of the bees using either model paints (Games Workshop, Nottingham, UK; Colony 1) or oil-based markers (Sharpie, Oak Brook, IL, USA; Colony 2). Neither marking method notably affected surface temperature readings in preliminary tests. Further preparations for surface temperature recordings included replacing the observation hives' glass windows with transparent polypropylene giftwrap (pbs-factory, Artikel 00347, Rheinland-Pfalz, Germany) and adjusting thermal camera settings (emissivity of honey bees 0.97–1.0, transmissivity of polypropylene 0.89). The giftwrap produced a nonlinear error when recording temperature as temperature increased, so absolute temperature measurements reported in this study have been adjusted. We calculated error by recording thermal images of a bee corpse through giftwrap and again without giftwrap, incrementally adjusting the bee's temperature by inserting a carbon film resistor within the body and controlling voltage with a transformer.

String stretched across both sides of the hive created a grid that was visible relative to the nest when thermally photographed. We mounted a thermal camera (FLIR S40 for Colony 1, FLIR SC660 for Colony 2, FLIR Systems Inc., Boston, MA, USA; accuracy ± 1°C or 1% of reading) on an adjustable, rolling monopod. We moved the camera from section to section of the grid and recorded data for each marked bee. We lined the hive and feet of our observation chair with dense foam to reduce substrate-borne vibrations. We also eliminated all ambient light. The hives were perpetually lit on each side with a desk lamp (Colony 1: 25 W, 230 V; Colony 2: Megaman, Compact 2000HPF 30 W, 4000 K) covered with red acetate filters (Colony 2: #27 Medium Red, transparency = 4%, peak at 670 nm, Supergel by Rosco, Stamford, CT, USA). Closer examination of behaviors was facilitated with a headlamp, also covered with the same red filter, selected because honey bees are reported to be less sensitive to frequencies beyond 600 nm [32] or 650 nm [33]. Although a preliminary test showed that bees could detect the filtered light sources, their behavior did not noticeably change if the bees were exposed to gradual changes in light intensity.

### Observations

Fifteen hours and 20 h after we collected bees for Colony 1 and 2 (3 h and 8.5 h after introduction into the hive, respectively), the bees were integrated into the colony, with no signs of aggression by other bees and no abnormal grooming. B.A.K. and M.S. systematically scanned for marked bees one section of the nest at a time, surveying bees every hour. As cell cleaners aged and became foragers, we recorded data for 24 consecutive hours during each of these two caste periods for Colony 1 (2 and 24 June 2006). As cell cleaners aged and became nurse bees, then food storers, we recorded data for 6 daytime hours and 6 nighttime hours during each of these three caste periods for Colony 2 (1000–1600 h and 2200–0400 h; 15, 18, and 26 August 2008). We began monitoring cell cleaners on the 1^st^ day after they eclosed, nurse bees on the 4^th^ day, food storers on the 12^th^ day and foragers on the 23^rd^ day (Fig. S1). We selected dates based on typical age-related caste determination in workers [34], [35].

We recorded thermal images of marked bees by pointing at each bee with soft forceps (marked with a pointer on one end to distinguish orientation of the head) as the thermal camera automatically recorded images every second. We verbally recorded each bee's behavior and identity (Olympus VN-4100PC Digital Voice Recorder, or audio track of Sony Handycam DCR-HC65, Tokyo, Japan), and confirmed identity of bees with a dim, handheld LED light. Due to temporary camera malfunction, we were unable to report data for nurse bees or food storers in Colony 1. To supplement the declining number of marked workers as Colony 1's bees became foragers, we captured and marked ten additional foragers of unknown age returning to the entrance of the hive.

We transcribed bee caste, individual identity, and behavior data from voice-recorded notes. Behavior included different sleep states, distinguished from wakeful activity by a bee's relaxed immobility and discontinuously ventilating abdomen. We examined each bee for 3–5 sec to determine her behavior. If she was potentially asleep inside or outside a cell we examined her for discontinuous ventilation, marked by a minimum of ten seconds without visible anterior-posterior abdominal pumping motions. Our ten-second pause between abdominal pumping bouts was based on measurements of honey bees inside cells made by Kleinhenz et al. [28], which appear to be shorter than the average respiratory pause measured by Kovac et al. [36] in isolated bees in metabolic chambers. If outside a cell, we reported antennae as immobile (deep sleep), or exhibiting swaying motions or minute twitches (light sleep) [27], [37], [38]. Reduced antennal mobility correlates with higher response thresholds, and antennal immobility for extended periods may be suggestive of a deeper sleep state [21].

**Figure 2:**
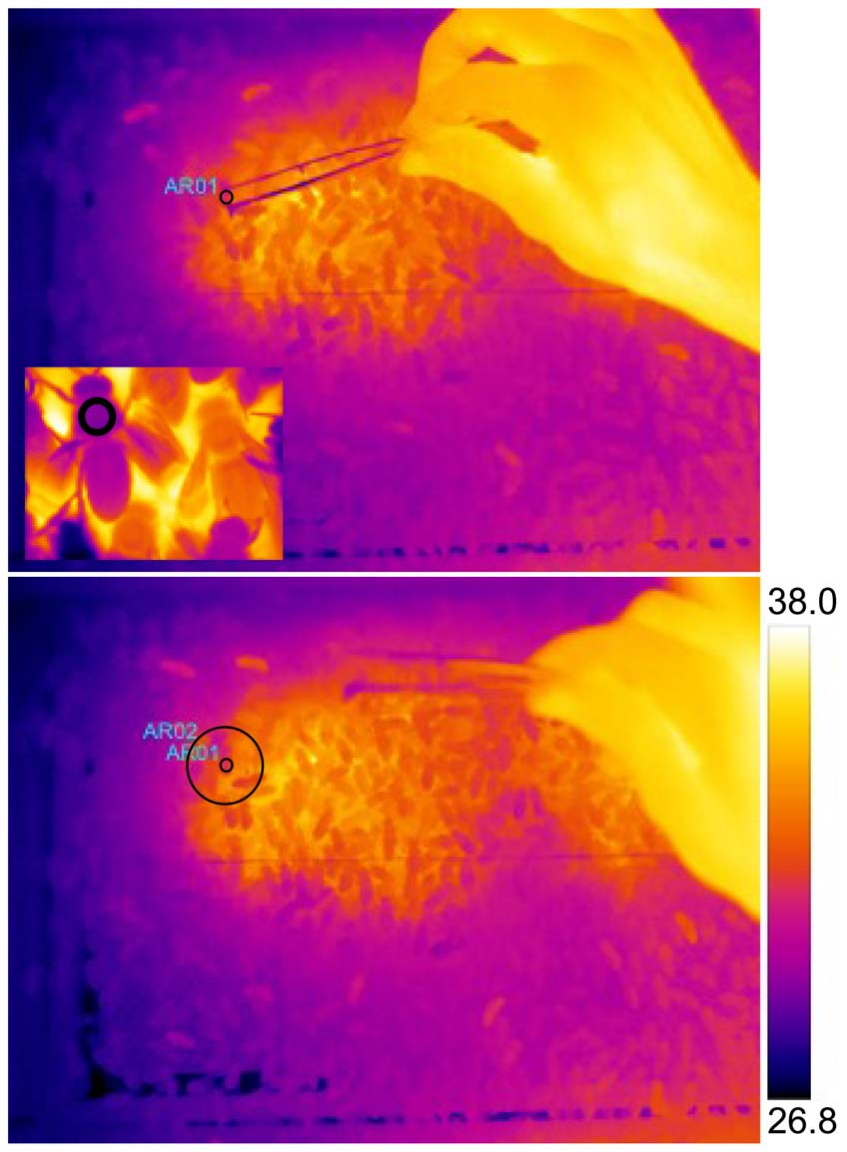
Collection of temperature data from a sequence of infrared images. The forceps were held open to encompass the heat-generating thorax of the bee (top) and camera software computed the average temperature within the selected region, represented by a circle in the inset image. For recording the surface temperature surrounding a bee (T_surr_), forceps were removed (bottom) so as not to influence the measurement, taken as the average temperature within the larger circle (radius  =  bee body length). T_diff_ is the difference of the average temperature within the large circle (T_surr_) from the average temperature in the smaller circle (T_th_). Temperature scale values (°C) were adjusted for thermal camera settings.

We transcribed a bee's location, or calculated it from thermal images. Infrared images were relayed to a computer and analyzed using camera-specific software (FLIR Systems ThermaCAM Researcher Professional software version 2.9). We recorded the average surface temperature of a bee's thorax (T_th_) and the average surface temperature of her surroundings (T_surr_), taken as the average temperature within a circle with the radius of one bee body length (Fig. 2). We report the difference of the two to indicate the temperature of the bee relative to the surface temperature of her surroundings (T_diff_). T_surr_ included the surface temperature of wax comb with a range of contents, wood from the hive frames, or bees; when calculating T_surr_, we did not exclude the area in which the examined bee appears. We also mapped the contents of comb cells for Colony 1 within 24 h of each census (5 and 25 June 2006) so that we could analyze bee behavior and temperature with respect to placement in the nest, particularly with respect to uncapped brood. We manually labeled cell contents on hive windows, removed and scanned these windows, and colored the discreet comb contents with different colors in Adobe Photoshop v.7.0 (Adobe Systems, San Jose, CA, USA).

Over the course of the study, Colony 1 grew from 1500 to 2800 individuals. Colony 2 housed about 2000 bees, and apparently lacked brood. Room air temperature varied moderately during study recording sessions (2006: 25.9°C, range = 24.7–26.6°C; 2008: 28.2°C, range = 24.6–28.4°C). The sun rose ca. 0510–0554 h and set ca. 2045–2130 h (CEST). For purposes of this study, daytime is defined as 0600–2200 h and nighttime as 2200–0600 h (CEST) to approximate ambient light (sunrise to sunset) conditions. No bees were observed to prematurely forage and by observing cell contents, we confirmed caste identity of several nurse bees and food storers.

### Analysis

All analyses are based on a total of 84 h of audio data and 78 h of thermal data (cell cleaners: 32 h, nurse bees: 12 h, food storers: 10 h, foragers: 24 h). Data include bee identity, behavior, position relative to the nest's perimeter (x, y coordinates), cell contents below bee, T_th_, and T_surr_ (Raw Data S1). Analyses computing placement in the nest were conducted using Python (version 2.6, http://www.python.org). Results from tests using continuous response variables and behavior as a categorical, independent variable are products of linear mixed-effects models, programmed in R [39], with bee group (caste), T_surr_, T_diff_, and position in the nest as fixed effects, and individual bee identity as a random factor (i.e., observations were nested within bee; Models S1). Linear mixed-effects models were fit using the lmer function in the lme4 package [40]. We used the multcomp package to perform likelihood-ratio tests to distinguish between competing models [41]; because of the complicated and unbalanced nature of the data (e.g. missing data, correlated covariates), we could not run standard likelihood-ratio tests [42]. We performed binary logistic regression using R, with T_surr_ as continuous predictor and behavior as the dependent variable. Means ± standard error means (s.e.m.) reported throughout the text and figure legends were calculated from averaged values, one value per bee. We set alpha at 0.05 and report two-tailed *P*-values for all tests.

## Results

**Figure 3:**
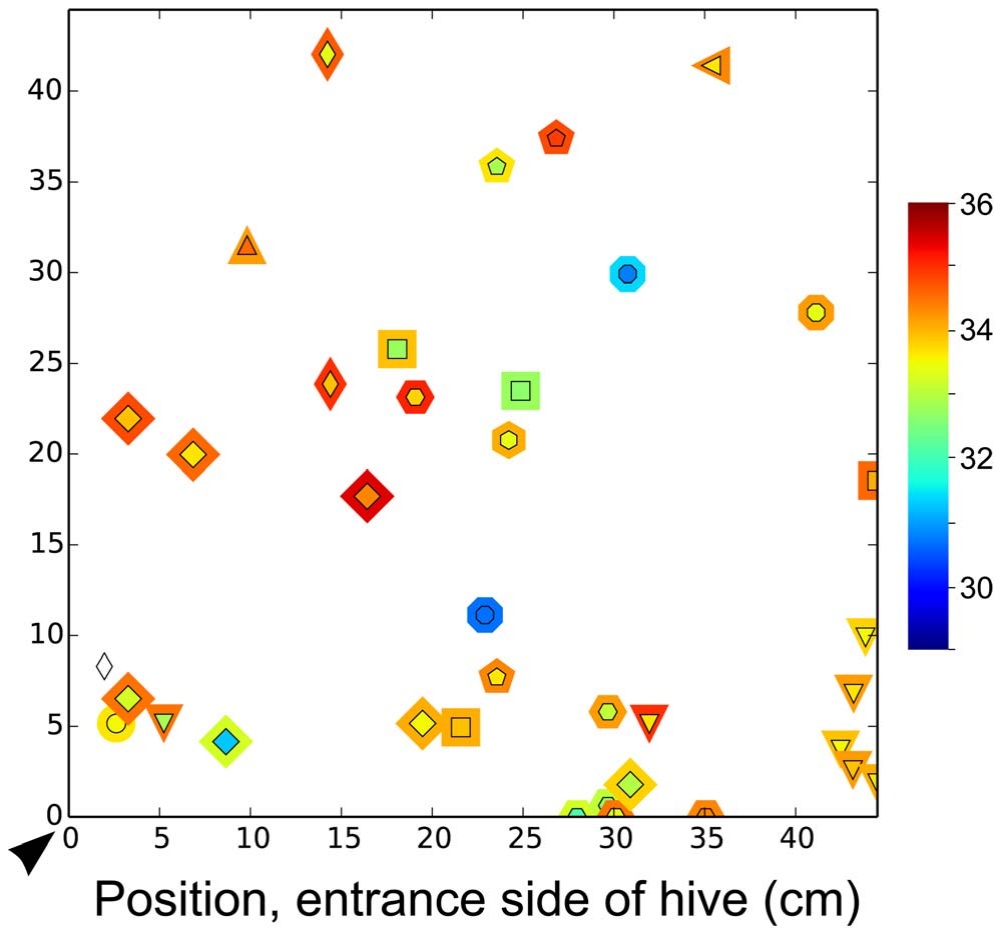
Sleep positions of individual foragers with respect to temperatures T_th_ and T_surr_. Unique shapes (including uniquely oriented shapes) distinguish different foragers (e.g., every triangle facing down represents data from one individual at different times, while every triangle facing up represents data from a different individual, squares  =  a third individual, etc.). Shapes represent T_th_ (inner shape) and T_surr_ (outer shape) for each observation of a marked, sleeping forager over 24 h on the entrance side of the hive (she may have also slept on the reverse side of the hive, as pictured in Fig. S3). Temperatures (°C) correspond with the color scale (white  =  no data, represented by diamond at lower left). Hive entrance/exit is indicated by arrowhead, and was restricted to one side of the hive. All relevant forager data are included in graph (*n* = 11 bees), but we treated forager as a random factor in mixed effects analyses to statistically cope with repeated measures of individuals. See Figs. S2 and S3 for differences across behaviors and worker castes.

**Figure 4:**
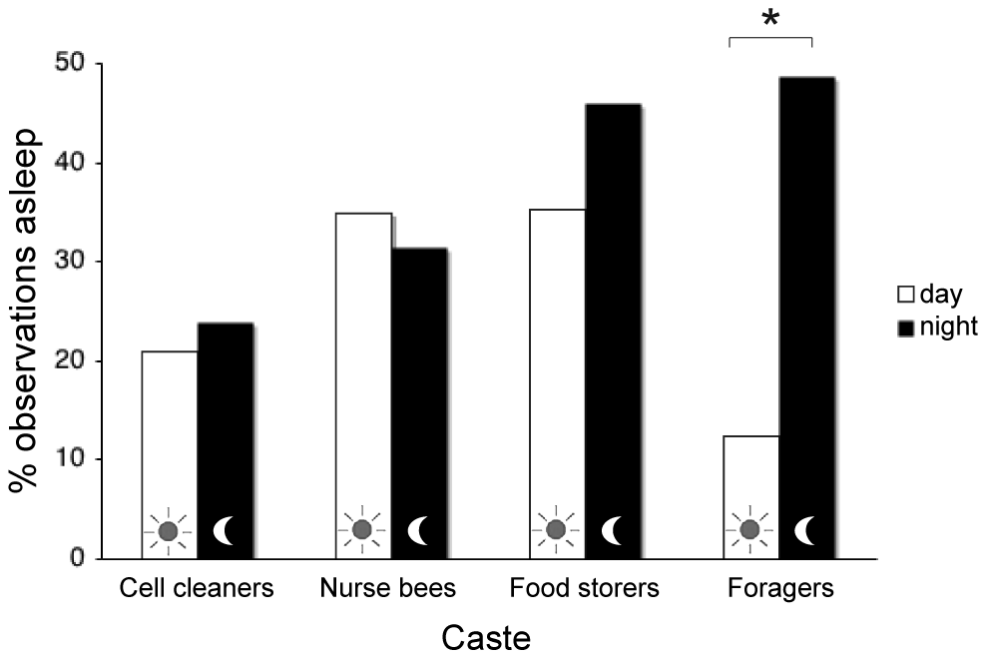
Proportion of observations bees of different worker castes were asleep during the day and night. Younger castes slept with no distinction between day and night. The clearest statistical distinction (signified with an asterisk, applying a linear mixed-effects model) appeared in foragers, with more time spent asleep during the night.

Honey bees belonging to all worker castes exhibited sleep (see Materials & Methods for operational definition). Cell cleaners slept inside cells, while each subsequent age caste slept less and less inside cells, with foragers having slept exclusively outside of cells (Table 1). Individual bees slept in many different areas over the course of a 24-h period (Fig. 3), but different worker castes consistently slept in different areas of the nest relative to position of brood comb and surrounding temperature, T_surr_ (see below). The only caste to clearly exhibit day-night periodicity with regard to their sleep was the foraging caste, with more total sleep at night (*z* = −6.05, *P*<0.00001; Fig. 4) and more deep sleep at night than during the day (*z* = −2.32, *P* = 0.038; *n* = 199 observations, 32 bees).

**Table 1:** Percent and total number of observations honey bees of different worker castes engaged in wakefulness and sleep.

	Worker castes							
Behavior	Cell cleaners (*n* = 95 bees)		Nurse bees (*n* = 47 bees)		Food storers (*n* = 43 bees)		Foragers (*n* = 32 bees)	
Light sleep	0.0%	0	6.4%	32	8.6%	31	12.0%	24
Deep sleep	0.1%	1	6.8%	34	23.9%	86	14.6%	29
Sleep inside cells	21.9%	196	19.5%	97	7.5%	27	0.0%	0
Total sleep	22.0%	197	32.7%	163	40.0%	144	26.6%	53
Awake	78.0%	697	67.3%	335	60.0%	216	73.4%	146
Total	100%	894	100%	498	100%	360	100%	199

Percent of sleep inside cells decreased with age and caste (99.5% of sleep for cell cleaners, 59.5% for nurse bees, 18.8% for food storers, and 0% for foragers; numbers in table represent percent of total observations, including when bees were awake). All observations for each bee are included.

### Sleep and proximity to the perimeter of nest

Our first objective was to calculate a bee's position relative to the perimeter of the comb and correlate this distance to a bee's behavior. Worker bees could position themselves anywhere between the perimeter of the comb (0 cm) and the center of the comb (22.25 cm from the nearest edge), the center being where brood are typically tended.

**Figure 5:**
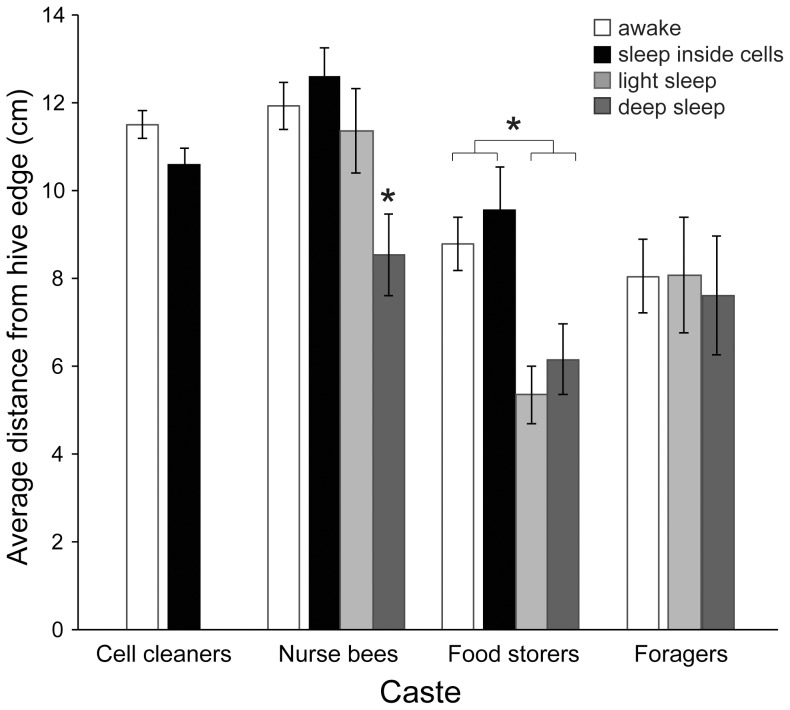
Distance from the nest's perimeter with respect to behavior and caste. Younger castes (cell cleaners and nurse bees) slept closer to the center of the nest (i.e., farther from the perimeter) than older castes (food storers and foragers). Bees slept outside cells closer to the perimeter of the nest than when they slept inside cells or were awake, except in the case of foragers, who were active during the day near the nest entrance. Cell cleaners did not sleep outside cells and foragers did not sleep inside cells, hence the absence of relevant bars. These data represent averages for castes calculated from average values per bee (± s.e.m.). Asterisks signify statistically significant differences within castes.

When worker bees slept inside cells, they tended to be at the same distance from the perimeter as when awake (e.g., Colony 2 cell cleaners: *z* = 1.41, *P* = 0.20, *n* = 158 observations of 44 bees), although cell cleaners in Colony 1 may have been slightly closer to the perimeter when asleep than when awake (*z* = 2.10, *P* = 0.050, *n* = 413 observations, 47 bees) (Fig. 5, S2).

When worker bees slept outside of comb cells, they tended to do so closer to the perimeter of the nest than when awake or sleeping inside cells (Fig. 5). Nurse bees exhibited light sleep at the same distance from the perimeter of the nest as when they were awake or sleeping inside cells, but they exhibited deep sleep when they were closer to the perimeter of the nest (*z* = 3.94, *P* = 0.0001; *n* = 469 observations, 47 bees) (Fig. 5, S2), and when nurse bees became food storers, both light and deep sleep were exhibited closer to the perimeter than when they were awake or sleeping inside cells (*z* = 4.43, *P*<0.00001; *n* = 332 observations, 43 bees) (Fig. 5, S3). Unexpectedly, foragers were not closer to the nest's perimeter when asleep than when awake. See possible explanations for this in the Discussion. Food storers and foragers spent more time both asleep and awake closer to the perimeter of the nest than younger castes (asleep: *F*_3,158_ = 27.87, *P*<0.0001; awake: *F*_3,204_ = 19.77, *P*<0.0001, ANOVA of pooled data), but no caste spent time closer to the perimeter with respect to day vs. night.

### Sleep position relative to surrounding temperature

Temperatures, particularly away from brood comb, fluctuate in regions of the nest, so we investigated the thermal position of bees with respect to day vs. night, as well as with regard to behavior. Our objective was to determine if differing thermal environments within the nest could serve as a predictor of a bee's behavior. We performed a binary logistic regression analysis to test the probability of predicting a bee's behavior by her T_surr_.

**Figure 6:**
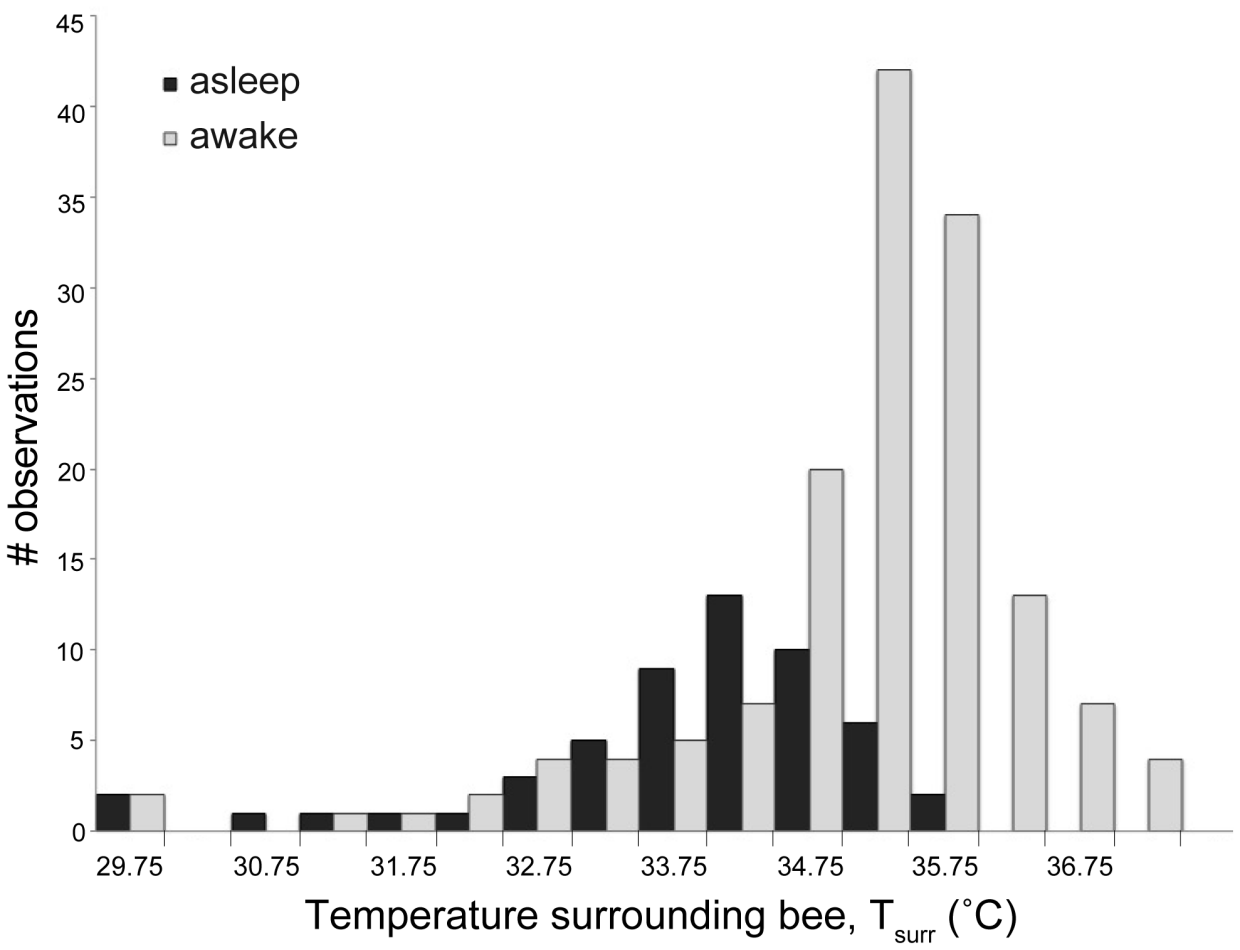
Surface temperature surrounding foragers as a continuous predictor of foragers' behavior. When T_surr_ was lower, foragers tended to be asleep; when T_surr_ was higher, foragers tended to be awake. Temperatures represent bins of +/−0.25°C (e.g., 29.75°C = 29.50–29.99°C; the unlabeled 30.25°C = 30.00–30.49°C). All data were collected from foragers outside cells. Food storers also exhibited lower T_surr_ when asleep than when awake.

When cell cleaners and nurse bees were asleep inside cells, they were in warmer regions of the nest than when they were awake (cell cleaners: 34.9±0.1°C vs. 34.3±0.1°C, *n* = 68 & 189, respectively; *z* = −2.89, *P* = 0.004). Nurse bees occasionally slept outside cells, and did so in regions not significantly thermally different from the regions they were in when awake (inside: 33.4±0.4°C, outside: 32.2±0.5°C vs. awake: 32.5±0.1°C, *n* = 51 & 115). The regions in which food storers slept were colder than the regions in which they were awake (asleep 31.6±0.2°C vs. awake 32.2±0.1°C, *n* = 31 & 63; *z* = 2.82, *P* = 0.005), but only when sleeping outside cells (31.5±0.2°C), not when sleeping inside cells (31.8±0.3°C). Likewise, the regions in which foragers slept (always outside cells) were colder than the regions in which foragers were awake (asleep 33.9±0.2°C vs. awake 35.1±0.1°C, *n* = 53 & 145; *z* = 5.03, *P*<0.00001) (Fig. 6).

Applying the same binary logistic regression analysis, we examined T_surr_'s ability to predict if a bee was observed during the day or night. Cell cleaners and nurse bees spent more time with warmer surroundings during the night than during the day (cell cleaners: 34.8±0.1°C vs. day 33.9±0.2°C, *z* = 4.47, *P*<0.00001; nurse bees: 33.0±0.2°C vs. day 29.4±0.5°C, *z* = 4.80, *P*<0.00001). Food storers, however, did not spend more time in colder or warmer regions of the nest (31.9±0.2°C vs. day 32.1±0.1°C, *z* = −0.95, *P* = 0.34) and foragers spent more time in colder regions of the nest at night than during the day (34.0±0.1°C vs. 35.3±0.1°C, *n* = 77 & 121; *z* = −5.72, *P*<0.00001).

### Sleep temperature

We recorded the average temperature of the dorsal surface of a bee's thorax (T_th_) and took the difference of T_surr_ from T_th_ as a measure of a bee's temperature relative to her surroundings (T_diff_) to test if variation in T_th_ or T_diff_ is explained by a bee's behavior. All data were extracted from bees when they were exposed (i.e., not inside cells).

**Figure 7:**
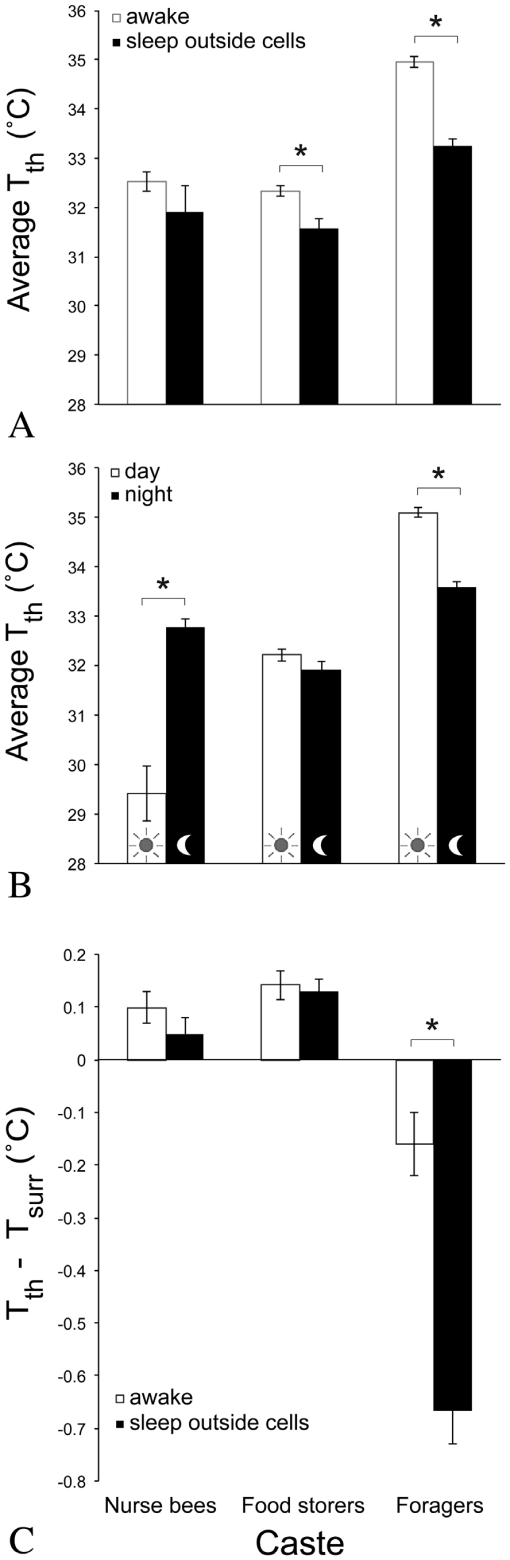
Average thoracic surface temperature of worker honey bees (T_th_) with regard to caste, behavior, day vs. night, or T_surr_. All measurements were taken from worker bees outside of cells; cell cleaners slept exclusively inside cells, so are excluded. Data represent averages for castes (± s.e.m.). Asterisks signify statistically significant differences within castes. (A) Average thoracic surface temperature of bees (T_th_) awake vs. asleep, (B) day vs. night, and (C) awake vs. asleep relative to surrounding surface temperatures (T_diff_).

Food storers were colder when in deep sleep than when awake (31.6±0.2°C vs. 32.3±0.1°C; deep: *z* = −3.22, *P* = 0.004; light: *z* = −2.25, *P* = 0.070; *n* = 83 observations, 36 bees). Likewise, foragers were colder when in either light sleep (33.0±0.28°C, *z* = −6.55, *P*<0.00001) or deep sleep than when awake (33.4±0.1°C vs. 35.0±0.1°C, *z* = −5.55, *P*<0.00001, *n* = 199 observations, 32 bees). But in nurse bees, T_th_ did not differ between light and deep sleep than when awake (*z* = −0.73 & 0.30, *P* = 0.84 & 0.99, light & deep sleep, respectively; *n* = 133 observations, 42 bees) (Fig. 7A).

**Figure 8:**
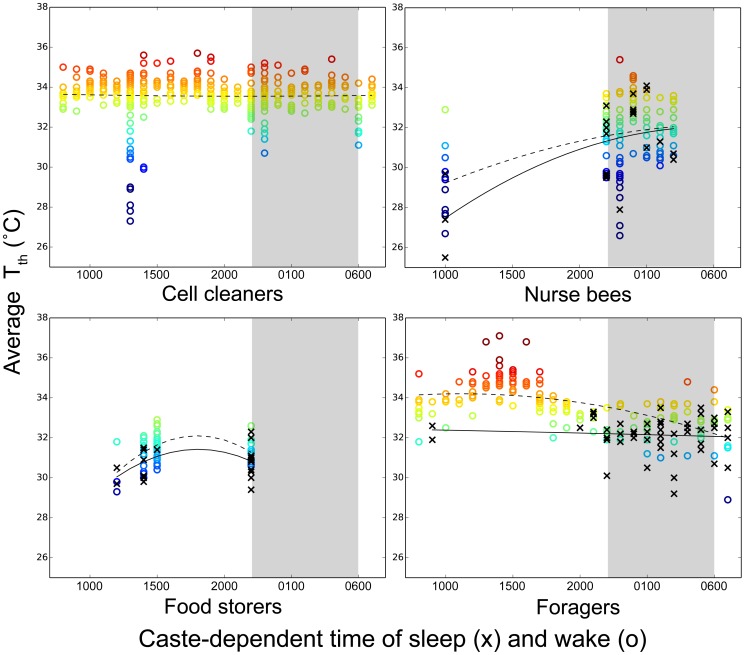
Average thoracic surface temperature of honey bee workers (T_th_) over the course of 24 h with respect to caste and behavior. An observation of an awake bee is represented by o with dashed lines fitting the data; an observation of a sleeping bee is represented by x with solid lines fitting the data. Gray backdrop represents nighttime and colors correspond with the temperature scale on y-axes and with Figs. 3, S2, and S3. Average T_th_ is reported per bee per census period and, although all bee data are included in these graphs, we treated bee as a random factor in mixed effects analyses. All measures were taken from bees outside of cells; no data exist for cell cleaners sleeping outside cells, hence the absence of x in the cell cleaner graph.

Day and night had an effect on some of the bees' T_th_. Nurse bees were warmer at night than during the day (32.8±0.2°C vs. 29.7±0.5°C, *z* = 5.89, *P*<0.00001; *n* = 133 obs., 42 bees). Food storers' T_th_ did not differ between day and night (32.2±0.1°C vs. 31.9±0.2°C, *z* = −1.36, *P* = 0.29; *n* = 83 obs., 36 bees) (Fig. 7B). Foragers were colder at night than during the day (33.6±0.1°C vs. 35.1±0.1°C, *z* = −8.08, *P*<0.00001; *n* = 199 obs., 32 bees). Day or night, foragers were colder (i.e., T_th_ was lower) when asleep than when awake (day: *z* = 4.87, *P*<0.00001, *n* = 121 obs., 27 bees; night: *z* = 3.54, *P* = 0.0008, *n* = 78 obs., 24 bees), and this colder sleeping temperature did not significantly differ between day and night (*z* = −1.58, *P* = 0.19; *n* = 53 obs., 16 bees). Sleeping food storers also showed no difference between day and night (*z* = 0.50, *P* = 0.81; *n* = 22 obs., 18 bees). In contrast, sleeping nurse bees were colder during the day than at night (*z* = 2.39, *P* = 0.023; *n* = 22 obs., 9 bees) (Fig. 8).

The only caste for which T_diff_ differed between sleeping and awake bees was the foragers. A sleeping forager's T_diff_ was more pronounced than an awake forager's (−0.66±0.06°C vs. −0.16±0.06°C, *z* = −4.08, *P*<0.001; *n* = 198 obs., 32 bees), particularly during deep sleep (−0.74±0.07°C) (Fig. 7C). Examples of T_diff_ in which T_th_ < T_surr_ are visible in Figs. 3 and S3.

All bees outside of cells were colder when situated closer to the perimeter of the nest, although foragers were colder specifically at night when closer to the perimeter of the nest (T∼day/night*proximity to perimeter; *z* = 2.79, *P* = 0.017; *n* = 199 observations, 32 bees). We could not examine sleep by cell cleaners with respect to their T_th_ or T_diff_ because cell cleaners slept inside cells, but awake cell cleaners' T_th_ and T_diff_ did not differ day vs. night (Fig. 8).

### Sleep relative to position of brood comb

**Figure 9:**
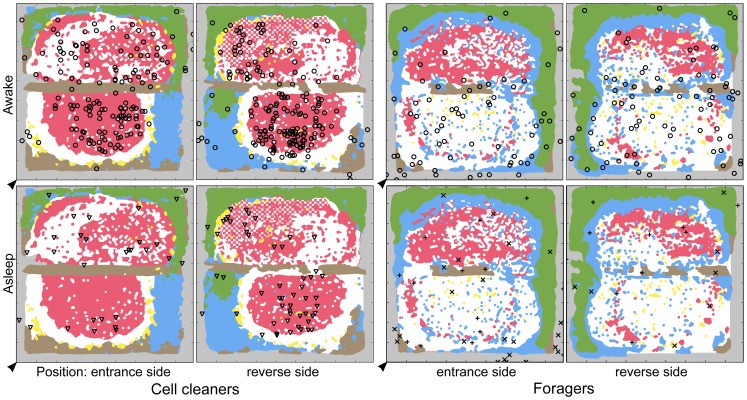
Caste-dependent maps of behavior with regard to comb cell contents. Cell cleaners (Colony 1 pictured) were awake (**o**) and asleep (inside cells; triangles) primarily in the brood comb area, spending more time asleep than awake with uncapped brood in their midst. Foragers, on the other hand, spent more time awake (**o**) than asleep (**x**  =  deep sleep, **+**  =  light sleep) near uncapped brood comb. White  =  uncapped brood, red  =  capped brood, yellow  =  pollen cells, blue  =  uncapped nectar, green  =  capped honey, brown  =  empty comb, gray  =  no comb. Nest entrance/exit is indicated by an arrowhead, and was restricted to one side of the nest.

Cell cleaners were more likely to be asleep than awake as the proportion of cells in their vicinity increasingly consisted of uncapped brood (surrounded by 32.27±0.03% vs. 22.56±0.03% uncapped brood, *t*_83_ = 2.14, *P* = 0.036; with trend in mixed effects model: *z* = −1.79, *P* = 0.074; *n* = 413 observations, 47 bees from Colony 1). After cell cleaners eventually developed into foragers, they were more likely to be awake than engaged in deep sleep as the proportion of cells in their vicinity increasingly consisted of uncapped brood (surrounded by 43.11±0.05% vs. 23.96±0.08% uncapped brood, *z* = 2.45, *P* = 0.027; *n* = 199 observations, 32 bees) (Fig. 9). We did not record cell contents for nurse bee and food storer stages.

## Discussion

Our objective was to describe caste-dependent sleep patterns in a honey bee colony, to produce “sleep maps” that reveal these patterns, and to discuss the patterns' functional significance. Sleep-site fidelity did not occur in individuals, contrary to what Clark and Gillingham [43] reported in anole lizards. However, spatial sleep patterns did occur among worker castes. The youngest workers (cell cleaners) were more often asleep than awake when surrounded by uncapped brood, while the opposite was true in the eldest workers (foragers) (Fig. 9). Workers slept less and less inside cells as they aged. Bees asleep inside cells were at the same distance from the perimeter of the nest as bees that were awake, while bees that slept outside cells did so closer to the perimeter of the nest than bees awake or asleep inside cells. Foragers appeared to sleep, and especially to exhibit deep sleep, on the periphery of the nest (Fig. S3), but two behaviors confounded this result: activity near the hive entrance (causing many awake bees to be near the perimeter), and sleep on wood frames running through the center of the nest. A two-frame observation hive is composed of one frame sitting above a second frame, creating an artificial break in the comb running through the center of the nest. Some bees were found sleeping on these centrally-located wood frames. Were proximity to the nest perimeter to be used to predict worker sleep in the future, forager activity near the hive entrance should be accounted for and all nest areas without comb (i.e., frames) should be treated as perimeters.

Why do honey bees sleep inside cells, why does this differ temporally among castes, and why is there a spatial distinction between sleep inside and outside cells within the same caste of bees? Sleep outside cells exposes bees to the arousing interactions of wakeful, mobile siblings ([27], supplementary video) and the greater density of bees found in the brood comb area could mean more frequent arousals and more fragmented sleep. By sleeping closer to the perimeter of the nest, exposed bees may increase their sleep. Consistent with this hypothesis, Klein et al. [27] reported increasing durations of uninterrupted sleep outside cells as bees aged/changed castes. Sleep inside cells may constitute an adaptive response to avoiding sleep fragmentation within the busy brood comb area. Younger bees slept more frequently inside cells than older bees and this could be the result of differential cell vacancy rates, with empty cells less frequently available in the brood comb, and more readily inhabited by bees already working in the brood comb area and within actual comb cells. Older bees may sleep less (food storers) or not at all (foragers) inside cells closer to the perimeter of the nest because they do not face the same degree of disturbance as they would if exposed in the brood comb. Sleep away from the brood comb may be a result of either learned or instinctual avoidance, or simple displacement due to repeated disturbance in the brood comb.

The temperature of a bee, of her surroundings, and the difference between the two appear to correlate with sleep behavior, offering specific opportunities for using temperature when identifying sleep across a colony of bees. We tested the probability of predicting a worker bee's behavior by her T_surr_ and found that sleep inside cells occurred either in areas as warm as when the bees were awake (nurse bees and food storers) or even warmer areas than when awake (cell cleaners). Sleep outside of cells occurred in colder regions than when awake (food storers and foragers), except in our small sample involving nurse bees. A bee can impact T_surr_, as best demonstrated by the actions of heater bees [28], [44]. A resting bee can be weakly endothermic [36], although when not heating, the effect of a bee's body on T_surr_ is less dramatic, or insignificant (Fig. 1A–C). It is worth noting that surrounding surface temperature can differ from ambient temperature experienced by a bee. This may account for the difference between our measure of T_surr_ encircling the average sleeping forager (33.8±0.2°C) and the preferred ambient temperature of sleep, as reported by Kaiser et al. [29] in isolated foragers (23–26°C, with extremes of 21 and 29°C) and Schmolz et al. [30] in isolated foragers (28°C, range: 26–29°C) and foragers within a nest (27.9°C, range: 23.8–30.8°C), or of worker *Bombus atratus* bumblebees [45]. On the other hand, our average measure of a forager's T_th_ during deep sleep (33.4±0.2°C) was congruent with measurements of resting bees inside cells (32.7±0.1–33.4±0.3°C) [28], and our lowest T_surr_ recordings are commonplace inside nests away from the brood area [21], [46].

We tested if T_th_ or T_diff_ is explained by a bee's behavior. T_th_ is lower when food storers and foragers are asleep than when they are awake and this lower T_th_ is statistically indistinguishable between day and night (Fig. 8). A relatively static lower sleeping temperature for the older castes is a consequence of sleeping closer to the colder periphery of the nest, although this correlation was confounded in foragers by their activity near the nest entrance. T_th_ of sleeping nurse bees did not differ from their wakeful T_th_ due either to insufficient sample size of nurse bees sleeping outside cells, or because sleep bouts are shorter in nurse bees than in older workers [27], not allowing for T_th_ to significantly decrease. Cell cleaners' T_th_ did not vary significantly when awake, likely due to the consistent warmth of the brood comb area within which cell cleaners in Colony 1 worked.

**Figure 10:**
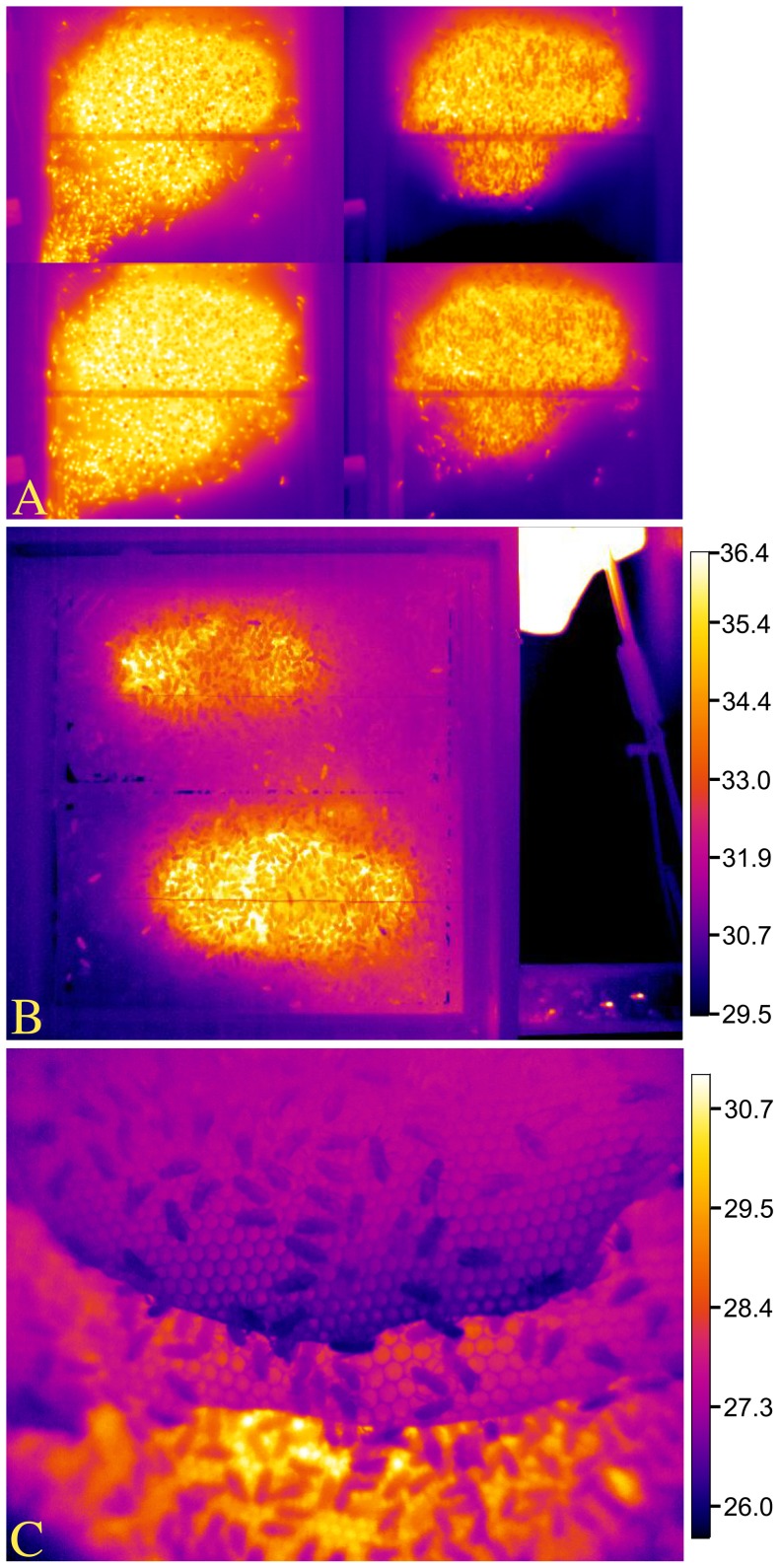
Infrared images revealing thermal activity across beehives. (A) Sequence of colony-scale changes across the entrance side of Colony 1. In clockwise order from the upper left corner, 1700, 0400, 0900 and 1500 h, respectively. Entrance/exit is in the lower left corner of the hive, leading out tube at left of each image. Brood comb is most easily seen as the glowing warm area at 0400 h. (B) Observation hive containing Colony 2, with filter-covered lamp at upper right, and bees visibly exiting hive tunnel at lower right. (C) Exposed nest composed of parallel sheets of comb, set up by Dirk Ahrens-Lagast to induce bees to construct a more natural nest architecture; not used in study. B.A.K. took all images with FLIR thermal cameras on non-experiment days under different ambient temperature conditions. Temperature scale values (°C) were adjusted for thermal camera settings (see Materials and Methods).

Brood comb is warmer and the bees are typically more active than in other regions of the nest, setting the stage for brood comb location to impact sleep positioning. We found the proportion of uncapped brood comb in the vicinity of cell cleaners and foragers correlated with behavior: cell cleaners in Colony 1 (Colony 2 had no brood) tended to be asleep more often and foragers less often when surrounded by uncapped brood. Brood comb is often centrally located, but can vary across observation hives (Fig. 10A,B), and the organization of brood comb and of thermal microclimates in an observation hive will differ from that of the three-dimensional hive box [47], [48] or architecture of a feral colony's nest. Observation hives are typically two-sided, but they are not as three-dimensional as natural nests, which consist of a series of parallel combs (Fig. 10C). Recording undisturbed behavior between the parallel combs of a more naturalistic hive (e.g., www.hobos.de), would be necessary to establish any similarities or differences between what we observed in observation hives and what may be occurring in more natural, three-dimensional nests.

Caste-dependent sleep patterns may be the consequence of selection pressures for sleeping in warmer or cooler areas. For example, honey bees may experience a trade-off between the benefits of warmth and obtaining unfragmented sleep, with more heat lost when alone [49]. By sleeping in colder areas, food storers and foragers may conserve energy, and by sleeping in warmer areas, cell cleaners may increase neural development or facilitate consolidation of memories. Schmolz et al. [30] reported that foragers sleep ectothermically and hypothesized that foragers select cool, but not maximally cool regions to sleep for the purpose of conserving energy while still promoting regenerative processes during sleep. Stabentheiner et al. [50] reported that ectothermy is most common in the youngest bees (0 to ∼2 d) and proposed that visitation to warm cells within the brood comb serves to increase flight muscle development. Additional explanations could include reduction of pathogen spread by segregation of sleeping castes, or an increased protection of younger, less expendable bees at the center of the nest. Alternatively, an awake bee may simply fall asleep without changing her location, or change location due to non-sleep-related reasons.

The observed caste-dependent patterns of sleep were consistent with previous studies of honey bees, including sleep inside and outside of cells [27], day-night periodicity of sleep in foragers [21], [27], [51] and the absence of periodicity in the younger castes (cell cleaners and nurse bees) [27], [52], [53], [54]. Food storers did not exhibit day-night periodicity in this study, but have previously been reported to exhibit either circadian sleep [27] or, in the case of a single subject, ultradian periodicity (12 h sleep-wake cycles) [52]. Future investigations, including testing our predictions to map spatial and temporal sleep behavior in colonies of honey bees, may add insight about functional attributes of sleep in a social setting.

## Supporting Information

Figure S1 **Timeline of data collection (black bars on timelines) for both Colony 1 and Colony 2.** We scheduled census times to fall within periods distinguishing the age-based worker castes. The beginning of the timeline represents eclosion, or the start of adulthood.
(TIF)

Figure S2 **Position of cell cleaners and nurse bees with respect to behavior and temperatures T_th_ and T_surr_.** Concentric circles represent T_th_ (inner circle) and T_surr_ (outer halo) for each honey bee observation. Temperatures (°C) correspond with the color scale at lower right (white  =  no data). Hive entrance/exit is indicated by an arrowhead, and was restricted to one side of the hive. All bee data are included in these graphs, but we treated bee as a random factor in mixed effects analyses to statistically cope with repeated measures of individual bees. Note that cell cleaners slept exclusively inside cells, so no T_th_ data were available for sleeping bees.
(TIF)

Figure S3 **Position of food storers and foragers with respect to behavior and temperatures T_th_ and T_surr_.** Concentric circles represent T_th_ (inner circle) and T_surr_ (outer halo) for each honey bee observation. Temperatures (°C) correspond with the color scale at lower left (white  =  no data). Hive entrance/exit is indicated by an arrowhead, and was restricted to one side of the hive. All bee data are included in these graphs, but we treated bee as a random factor in mixed effects analyses to statistically cope with repeated measures of individual bees. Note that foragers exhibited wakeful activity near hive entrance, which eliminated average wake-sleep differences in distance from the nest perimeter. For a more focused look at the changing sleep sites of foragers, see Fig. 3.
(TIF)

Raw Data S1 **Raw data, used for analyses in R.** Caste: c  =  cell cleaner, n  =  nurse bee, fs  =  food storer, f  =  forager. Newforager: yes  =  forager added to supplement dwindling sample of foragers in Colony 1; this category was not used in analyses. d_n: d  =  day, n  =  night. Behav  =  a more specific set of behavioral categories than Beh. cm_edge  =  position of bee, in cm from edge of hive. UnderColor  =  the color of the hive map on which the bee is positioned (e.g., white signifies the region of the comb in which cells contain uncapped brood).
(CSV)

Models S1 **Models used in statistical analyses.** Models are written for analysis in R.
(R) Click here for additional data file.
