# Supplementary figures and images for: Mapping Sleeping Bees within Their Nest: Spatial and Temporal Analysis of Worker Honey Bee Sleep
Source: PLoS One. 2014 Jul 16;9(7):e102316. doi: 10.1371/journal.pone.0102316 (PMC4100802; doi:10.1371/journal.pone.0102316)

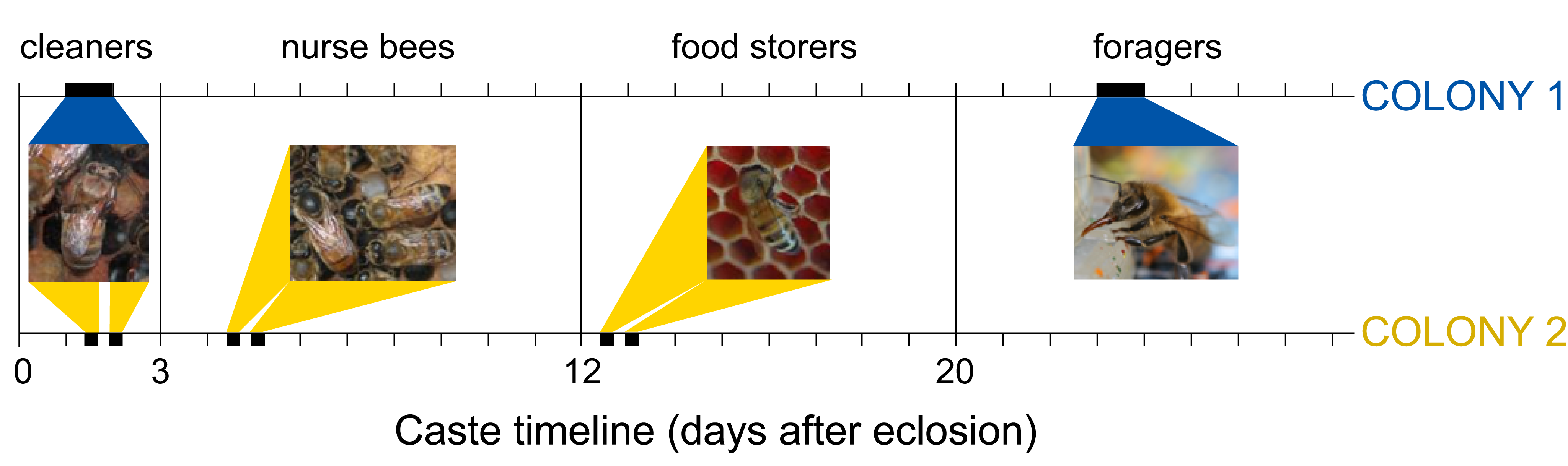

Supplement: Figure S1 — Timeline of data collection (black bars on timelines) for both Colony 1 and Colony 2. We scheduled census times to fall within periods distinguishing the age-based worker castes. The beginning of the timeline represents eclosion, or the start of adulthood. (TIF) [file pone.0102316.s001.tif]

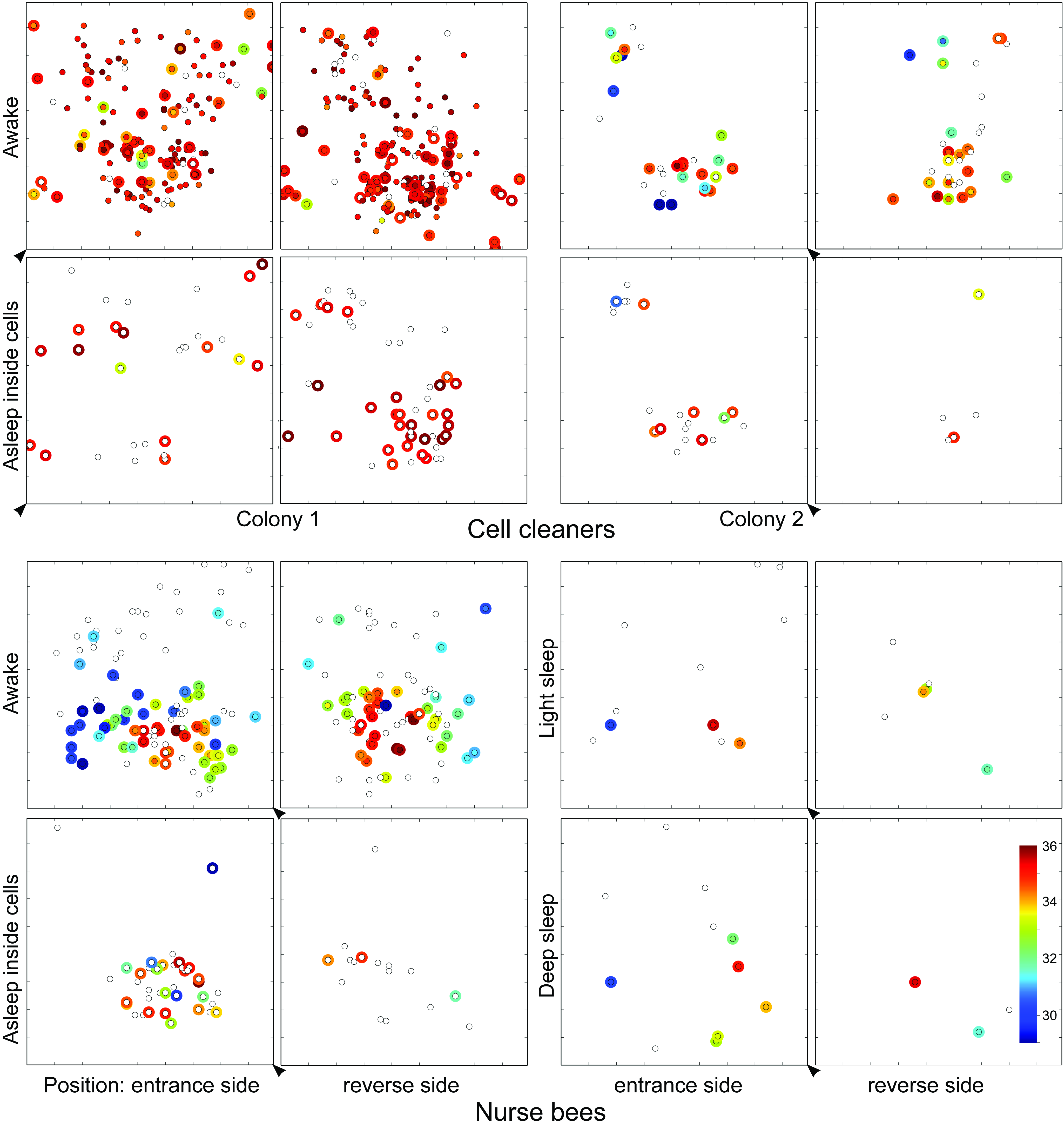

Supplement: Figure S2 — Position of cell cleaners and nurse bees with respect to behavior and temperatures Tth and Tsurr. Concentric circles represent Tth (inner circle) and Tsurr (outer halo) for each honey bee observation. Temperatures (°C) correspond with the color scale at lower right (white = no data). Hive entrance/exit is indicated by an arrowhead, and was restricted to one side of the hive. All bee data are included in these graphs, but we treated bee as a random factor in mixed effects analyses to statistically cope with repeated measures of individual bees. Note that cell cleaners slept exclusively inside cells, so no Tth data were available for sleeping bees. (TIF) [file pone.0102316.s002.tif]

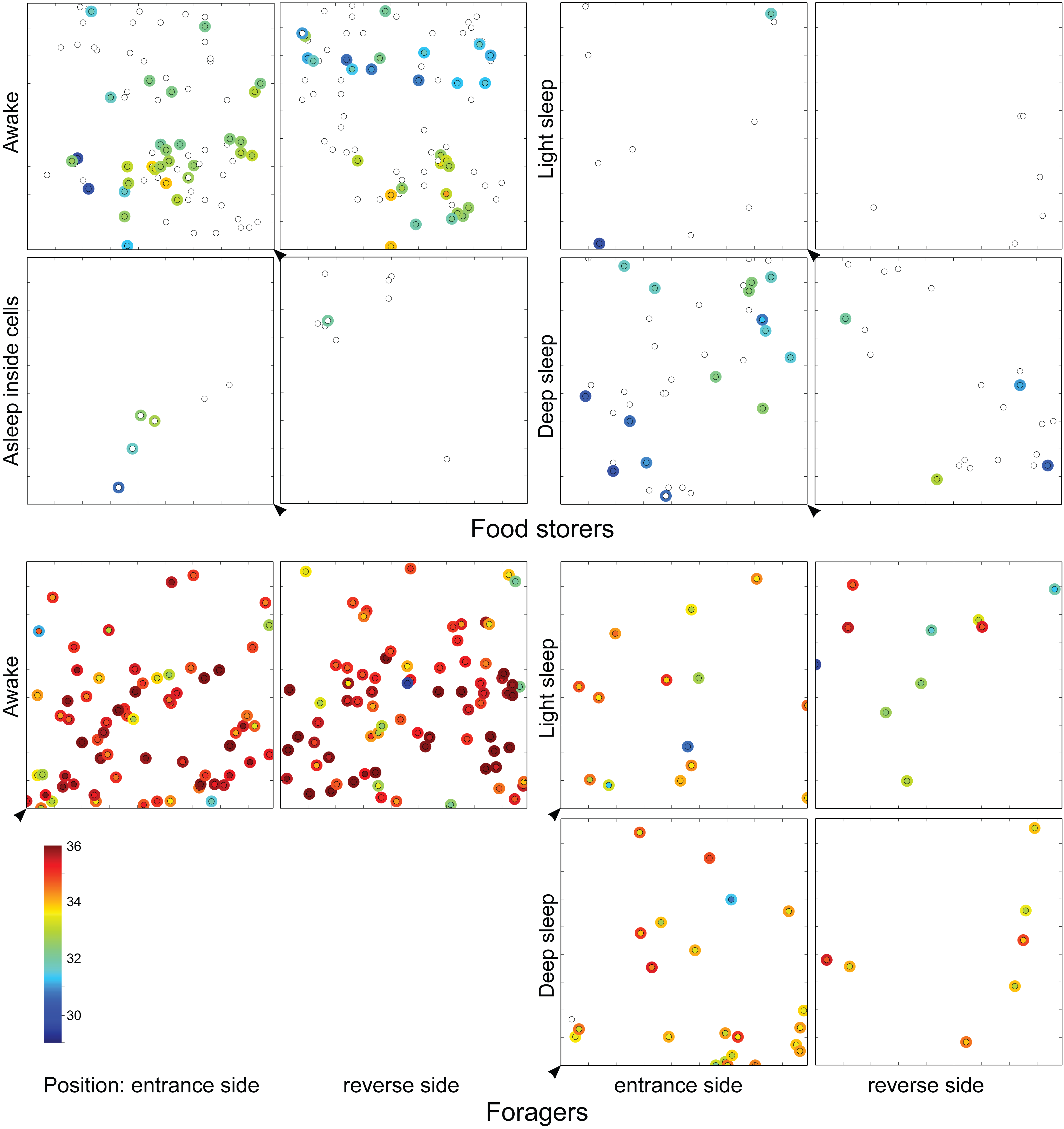

Supplement: Figure S3 — Position of food storers and foragers with respect to behavior and temperatures Tth and Tsurr. Concentric circles represent Tth (inner circle) and Tsurr (outer halo) for each honey bee observation. Temperatures (°C) correspond with the color scale at lower left (white = no data). Hive entrance/exit is indicated by an arrowhead, and was restricted to one side of the hive. All bee data are included in these graphs, but we treated bee as a random factor in mixed effects analyses to statistically cope with repeated measures of individual bees. Note that foragers exhibited wakeful activity near hive entrance, which eliminated average wake-sleep differences in distance from the nest perimeter. For a more focused look at the changing sleep sites of foragers, see Fig. 3. (TIF) [file pone.0102316.s003.tif]
